# Evaluation of ELISA and immunoaffinity fluorometric analytical tools of four mycotoxins in various food categories

**DOI:** 10.1186/s13568-023-01629-5

**Published:** 2023-11-03

**Authors:** Marina H. Boshra, Ghadir S. El-Housseiny, Mohammed M.S. Farag, Khaled M. Aboshanab

**Affiliations:** 1 Department of Mycotoxins, Central Public Health Laboratories (CPHL), Ministry of Health, Cairo, Egypt; 2 Department of Microbiology and Immunology, Faculty of Pharmacy, Ain Shams University, Cairo, 11566 Egypt; 3 Botany and Microbiology Department, Faculty of Science, Al-Azhar University, Cairo, 11884 Egypt; 4 Armed Forces College of Medicine (AFCM), Cairo, Egypt; 5 Department of Microbiology and Immunology, Faculty of Pharmacy, Ain Shams University, Abbassia, 11566 Cairo Egypt

**Keywords:** Aflatoxins, Ochratoxin A, Zearalenone, Deoxynivalenol, Fluorometer, Immunoaffinity

## Abstract

Mycotoxins (MTs) are secondary toxic metabolites that can contaminate food, impacting quality and safety, leading to various negative health effects and serious pathological consequences conferring urgent need to evaluate and validate the currently standard methods used in their analysis. Therefore, this study was aimed to validate ELISA and VICAM immunoaffinity fluorometric, the two common methods used to monitor the level of MTs according to the Egyptian Organization for Standardization and Quality Control. A total of 246 food samples were collected and tested for Aflatoxins (196 samples), Ochratoxin A (139), Zearalenone (70), and Deoxynivalenol (100) using both analytical methods. Results showed that aflatoxins exceeded limits in 42.9, 100, and 13.3% of oily seeds, dried fruits, and chili and spices, respectively. For ochratoxin A, 3.9% of Gramineae and 8% of spices and chili (locally sourced) exceeded the limits, while 17.6% of imported pasta and noodles exceeded the limits for deoxynivalenol. Significant differences for the aflatoxins and ochratoxin A detection among different categories of chocolate, dried fruits, and oily seeds (p-value < 0.05). No zearalenone contamination was detected in the exported, imported, and locally sourced categories. No deoxynivalenol contamination was detected in the tested Gramineae category. In contrast, for pasta and noodles, the imported samples exhibited the highest contamination rate (above the upper limit of 750 µg/kg) with 17.6% of the samples testing positive for deoxynivalenol with no significant difference among different sample categories of Gramineae, pasta, and noodles (p-value > 0.05). In conclusion, our study found no significant differences between the ELISA and immunoaffinity fluorometric analysis in the detection of the respective MTs in various food categories and therefore, they can substitute each other whenever necessary. However, significant differences were observed among different food categories, particularly the local and imported ones, highlighting the urgent need for strict and appropriate control measures to minimize the risk of MTs adverse effects.

**Supplementary Information:**

The online version contains supplementary material available at 10.1186/s13568-023-01629-5.

## Key Points

1. Screening and quantitative estimation of four major MTs in various food matrices to ensure food safety and quality have been done according to standard guidelines.
2. Our findings revealed that some of the tested MTs exceed limits and the possible attributed factors have been discussed and highlighted in the manuscript.
3. Results obtained proved non-significant differences between the ELISA and immunoaffinity fluorometric analysis in the detection of Aflatoxins, Ochratoxin A, Zearalenone, and Deoxynivalenol in various food categories.

**Supplementary Information:**

The online version contains supplementary material available at 10.1186/s13568-023-01629-5.

## Introduction

Food quality and safety are crucial to ensure that the market is not harmed by selling subpar or hazardous food. The biggest threat to the safety of human food is posed by food-borne microorganisms, followed by MTs. additionally, MTs constitute the greatest risk when it comes to animal feeds (Bankole and Adebanjo 2003). MTs are secondary metabolites produced by phylum Ascomycota of the Fungi kingdom (Chatterjee et al. 2023). They compromise a group of low molecular weight molecules with a wide range of chemical properties that can be found in processed foods, agricultural goods, and the environment (Chilaka and Mally 2020). They are regarded as one of the most significant pollutants in foods, and feeds. As stated by the United Nations Food and Agricultural Organization (FAO), more than 25% of the global agricultural production contains MTs, which causes financial losses in the grain business (Rahmani et al. 2009). MTs are mostly ingested, but they can also be inhaled through dermal and respiratory routes. The magnitude of harmful mycotoxin effects on human or animal health is primarily determined by the level of exposure (dosage and duration), mycotoxin kind, nutritional and physiological state, and potential synergistic effects of additional chemicals that the animals or humans are exposed (Gajecka et al. 2013; Bertero et al. 2018).

In 1960, the incidence of the AF-caused sickness, turkey X disease, which claimed the lives of over 100,000 turkeys, aroused interest in MTs. Following that, it was discovered that Hepatocellular carcinoma (HCC) is a cancer that is brought on by AFs in both humans and animals, which increased curiosity about MTs research (Liew and Mohd, 2018). These MTs are produced in several foods such as dried fruits, Gramineae (wheat, oat, rice, flour, corn, cereals, biscuits, cakes, breakfast cereals for pediatrics), chili, spices, nuts, coffee (roasted and green), chocolate, cocoa and milk (Chatterjee et al. 2023). More than 500 MTs have been identified as having toxic potential so far. These include the OTA and AFs generated by *Aspergillus* species, the trichothecenes and fumonisins produced by *Trichothecium* species, and the ZEN, DON, and fumonisins produced by Fusarium species (Horky et al. 2018). AFs, OTA, ZEN, DON, and fumonisins are the five most significant MTs (Omar et al. 2020). The four most significant AFs discovered are AFB1, AFB2, AFG1, and AFG2 relying on their UV fluorescence and relative chromatographic mobility during thin-layer chromatography. AFB1 is the most prevalent AFs found in human and animal feed. In fact, AFB1 is also the strongest known mammalian hepatocarcinogen and is categorized by the International Agency for Research on Cancer (IARC) as a Group I carcinogen (Liew & Mohd, 2018). AFB1’s primary target site is the liver. AFB1 undergoes biotransformation to become aflatoxin M1, which is hydroxylated, when cows eat AFs-contaminated feed (Kunter et al. 2017).

On the other hand, OTA’s primary target site is the kidney. OTA has been connected to the etiology of several renal illnesses, including chronic interstitial nephritis (CIN) in Tunisia, Balkan endemic nephropathy (BEN), and kidney tumors in various Balkan Peninsula endemic locations (Fuchs and Peraica 2005). Additionally, OTA is a nephrotoxic, teratogenic, and immunosuppressive substance that was categorized as a Group 2B probable human carcinogen by the IARC (Ladeira et al. 2017). On the other hand, ZEN is considered an estrogenic mycotoxin. In many animal species, particularly swine, cattle, and sheep, higher levels of ZEN intake (ZEN toxicosis) lead to increased estrogenic activity and cause disrupted conception, miscarriage, and other reproductive difficulties like infertility, vulval edema and the feminization of male animals (Elkenany and Awad 2020). Alternatively, DON is a vomitoxin that causes vomiting, nausea, digestive issues, oxidative damage, and reproductive toxicity in both animals and humans although it is not a human carcinogen (Ji et al. 2019). DON belongs to Group 3 according to the IARC (non-carcinogenic substances) (Ji et al. 2019).

Numerous factors, including climate conditions, pest infestations, and improper harvesting and storage techniques, have an impact on the proliferation of fungus as well as the formation of MTs in food. Mold grows more quickly, and MTs are produced as the humidity level rises during storage. Controlling the storage conditions is one approach to reducing mycotoxin formation (Omar et al. 2020; Magan and Olsen 2004). Studies on food contamination and exposure to MTs are realtively uncommon in the Middle East (Raad et al. 2014). Furthermore, MTs have proven toxic effects at very low concentrations, affecting the quality and safety of foods resulting in various acute and chronic toxicities and several health complications. Accordingly, there is an urgent need to detect, quantitatively measure, and continuously monitor the level of the respective MTs in various food matrices to ensure food safety and quality.

The development of numerous analysis methods for the detection and quantification of MTs in food samples has resulted from all of these attempts to set mycotoxin limits and standards (Janik et al. 2021). Numerous analytical techniques, and rapid strip screening tests, have been validated and used for the analysis of MTs in food (Alshannaq and Yu, 2017; Agriopoulou et al. 2020). For on-site mycotoxin investigation, quick and easy screening approaches include immunochemical methods like ELISA (Al-Jaal et al. 2019). Multiple samples can be tested simultaneously by ELISA, which also offers precise detection. Compared to chromatographic technologies like HPLC or TLC, it is a high-throughput test with lower sample volume requirements and fewer clean-up steps (Oplatowska et al. 2018; Singh and Mehta, 2020). however, This method has certain drawbacks. Antibodies can interact with substances that share similar chemical groups. Mycotoxin concentrations in test samples may be underestimated or overestimated because of matrix effect or matrix interference, which occurs in the ELISA method standards (Janik et al. 2021). Additionally, insufficient ELISA validation restricts the approach to the matrices for which it has been validated (Omar et al. 2020).

Regarding the fluorometric assay with immunoaffinity clean-up column. The MTs must be extracted from the matrix in order to be released. The extract must be thoroughly cleaned to minimize matrix effects and get rid of everything that might get in the way of the next mycotoxin detection. The extract can be made more selective and sensitive through purification, which also improves quantification accuracy and precision. Solid phase extraction (SPE) and immunoaffinity columns (IAC) are the most often used techniques for mycotoxin cleanup because they are effective, repeatable, and provide a wide range of selectivity (Alshannaq and Yu, 2017). IACs can be used as an effective, universal purification technique for tracing MTs since they are highly sensitive and selective. Furthermore, due to the specificity of the antibodies, it is a user-friendly and solvent-saving method (Liu et al. 2018). This approach does, however, have significant drawbacks. MTs have a limited capacity for absorption by columns, thus if the sample’s mycotoxin content exceeds that capacity, the mycotoxin will not be efficiently captured and bound, leading to incorrect results. In addition, the matrix’s many components may prevent the antibodies from working properly (Castegnaro et al. 2006). Organic solvents are another drawback since they might denature or devitalize antibodies, making it difficult to reuse IACs. Additionally, this technology has extremely high operational expenses (Liu et al. 2018). Also, it is time-consuming and involves the use of numerous harmful and toxic organic chemicals (Agriopoulou et al. 2020). Despite the enormous advancements made in this area, there are still many difficulties and drawbacks to these analytical techniques that need to be resolved. MTs’ chemical diversity, co-occurrence, varying amounts in agricultural products, and complex food matrices contaminated with MTs necessitate the use of specialized extraction, cleanup, and detection techniques (Hajslova et al. 2011). To comply with mycotoxin legislation and limits, to safeguard consumer health, and to promote agriculture, it is necessary to continuously develop mycotoxin analysis methodology (Stroka et al. 2016).

Therefore, the objective of this study was to evaluate the two standard commonly used techniques in Egypt, ELISA spectrophotometric (semiquantitative) and VICAM immunoaffinity column (IAC) fluorometric methods (quantitative) for the detection and estimation of four clinically relevant MTs in various food matrices and categories (local, imported and exported). The food samples were randomly collected from the Egyptian market and were based on standard screening protocol and sample size standard guidelines for detection and estimation of the levels of aflatoxins (AFs), Ochratoxin A (OTA), Zearalenone, and Deoxynivalenol.

## Materials and methods

### Sample collection and preparation

A total of 246 representative samples of various food samples and different categories (local (154), imported (73), and exported (19) were obtained from the Egyptian market in 2022 according to AOAC 977.16 (http://www.aoacofficialmethod.org/index.php?main_page=product_info&products_id=2065 (accessed on 20 August 2022) (Omar et al. 2020). The collected samples were tested for the presence of the four major MTs, including Aflatoxins (AFs; 196 samples), Ochratoxin A (OTA; 139), Zearalenone (ZEN; 70), and Deoxynivbyalenol (DON; 100) using the ELISA followed by immunoaffinity fluorometric analysis as previously reported by EOSQC standard guidelines (2010) (https://www.eos.org.eg/en/standard/12561, (accessed on 8 august 2023).

For lots weighing more than 50 tons, we took 100 incremental samples from the sub-lots to get an aggregate sample weighing 10 kg; but, for lots weighing less than 50 tons, we only took 3 to 10 incremental samples, depending on the lot weight, to produce an aggregate sample weighing 1 to 10 kg (Rahmani et al. 2009). To establish homogeneity, samples were transferred to the laboratory and then crushed (by means of sanitized food cutters) and mixed carefully by a horizontal shaker (Benchmark Scientific, Orbi Shaker, Edison, USA) to be prepared for subsequent analysis (Elkenany and Awad 2020).

### Screening of certain mycotoxins (AFs, OTA, ZEN and DON) using ELISA

#### Chemicals and reagents

Screening for the four different MTs was done using an ELISA Test kit (RIDASCREEN^®^, Manufacturer R- Bio pharm AG, Darmstadt, Germany). For AFs (Art. Nr. R4701), OTA (Art. No. R1312), ZEN (Art. No.: R1401), DON (Art. No.: R5906). All reagents required for the enzyme immunoassay, were included in the test kits. Ready to use standards were included in the test kits with concentrations 0 µg/L, 0.05 µg/L, 0.15 µg/L, 0.45 µg/L, 1.35 µg/L, 4.05 µg/L (1.3 mL each) for AFs; 0 µg/L, 0.03 µg/L, 0.1 µg/L, 0.3 µg/L, 1 µg/L, 3 µg/L (1.3 mL each) for OTA; 0 ng/L, 50 ng/L, 150 ng/L, 450 ng/L, 1350 ng/L, 4050 ng/L (1.3 mL each) for ZEN and 0 µg/L, 3.7 µg/L, 11.1 µg/L, 33.3 µg/L, 100 µg/L (1.3 mL each) for DON. diluted ECO extractor (10x concentrate) was included in the test kit of OTA to be used in extraction (dilution was done 1:10 with distilled or deionized water at 2 to 8 ℃). In the test kit of ZEN, Buffer 1 (50 mL) was included. A microtiter plate spectrophotometer was required for semi-quantification (screening). Special software, RIDASOFT^®^ Win.NET (Art. No. Z9996FF) was used in the screening process. Filter paper: Whatman No. 1 or its equivalent was purchased from VICAM (https://www.vicam.com/category/aflatoxin-testing-solutions (accessed on 12 August 2023). Methanol (HPLC grade, purity ≥ 99.9%) was purchased from Sigma Aldrich (Merck, Kga, Darmstadt, Germany). Ultra-pure water was purchased and products by the Milli-Q purification system (Milli-Q from Millipore, USA).

### Method of analysis for mycotoxins

#### Aflatoxins (AFs)

Ground samples (5 *g*) and 25 mL of 70% methanol were mixed for 10 min at room temperature by vortexing and filtered through a Whatman No. 1 filter or centrifuged (10 min / 3500 g / room temperature). Then, 100 µL of the filtrate/supernatant was diluted with 600 µL distilled water. The wells were then filled with 50 µL of standard or sample in duplicate together with 50 µL of the conjugate. Then, 50 µL of the antibody was added to each well, and the plate was gently shaken for mixing and then allowed to sit at room temperature (20 to 25 ℃) in the dark for 30 min. After incubation, the well contents were discarded and the microwell holder was tapped upside down strongly (three times) on absorbent paper. Then wells were washed with 250 µL wash buffer 3 times after which 100 µL of substrate/chromogen was added to each well, gently mixed by hand shaking the plate, and incubated for 15 min at room temperature (between 20 and 25 ℃) in the dark. The stop solution (100 µL) was then pipetted into each well followed by manual shaking of the plate. After 30 min, the extinction was determined at 450 nm.

### Ochratoxin A (OTA)

An aliquot of 10 gm ground sample was weighed and 50 mL of diluted ECO extractor was added followed by vortexing (10 s). The sample was then shaken violently for 5 min (either manually or with a shaker set to 420 rounds per minute) followed by centrifugation for 5 min at room temperature (20 to 25 ℃, 3500 *g*). Subsequently, 1 mL of the supernatant was diluted with 1 mL of wash buffer. An aliquot of 50 µL of standard or sample was then used to fill the wells in duplicate. Each well then received 50 µL of the conjugate which was gently combined by manually shaking the plate for 30 min at room temperature (20 to 25 ℃) in the dark. After that, the well contents were discarded, and the assay was continued as mentioned previously for AFs.

### Zearalenone (ZEN)

Aliquots of 5 *g* of ground samples were weighed into a suitable container and 25 mL of methanol (70%) were added. Then, vigorous shaking (either manually or using a shaker) was carried out for three min. The extracts were centrifuged (10 min/3500 *g*, room temperature) or filtered. Following that, sample dilution buffer (buffer 1) was used to dilute the filtrates or supernatants 1:7 (100 µL supernatant or filtrate + 600 µL buffer 1). Then 50 µL of standard or sample were used to fill the wells in duplicate. The ZEN enzyme conjugate (diluted 1:11 in buffer) was added in 50 µL portions to each well. After that, the plate was gently stirred by handshaking and incubated for 2 h at room temperature (20 to 25  ℃) in the dark. After that, the well contents were discarded, and the assay was continued as mentioned previously for AFs.

### Deoxynivalenol (DON)

Aliquots of 5 *g* of ground samples were weighed into a suitable container and 25 mL of distilled water was added and shaken for three min. Whatman No. 1 filter was used to filter the extract. Then 50 µL of standard or sample were added to the wells in duplicate. Then each well received 50 µL of the conjugate. Subsequently, each well received 50 µL of the anti-DONantibody, which was carefully mixed by hand shaking the plate and incubated for 30 min at room temperature (20 to 25 ℃) in the dark. After that, the well contents were discarded, and the assay was continued as mentioned previously for AFs.

### Quantitative determination of certain mycotoxins (AFs, OTA and ZEN) using VICAM

#### Aflatest, Ochratest, zeralatest immunoaffinity column (IAC) followed by fluorometric method

##### Chemicals and reagents

AflaTest Columns (25 per box), OchraTest Columns (25 per box), zearalaTest Columns (25 per box), Microfiber Filters, filter paper Whatman no.1, 1.0 m, 9 cm (100), Tween-20 (50 mL), 10X Concentrate of 0.01% Tween/PBS (150 mL), 5X Concentrate of 2% Tween/PBS (300 mL), 10X Concentrate of 0.1% Tween/PBS (150 mL), 10x concentrate of PBS (Phosphate Buffered Saline), AflaTest Developer (50 mL), OchraTest Eluting Solution (50 mL)and Zearalatest developer were purchased from VICAM A., WATERS, USA. Methanol (HPLC Grade, Acetonitrile HPLC Grade), ACS Grade Salt (100 *g*) (nonionized salt, NaCl) Zinc acetate powder, and AlCl3 powder were purchased from Sigma Aldrich. A commercial Blender with a Stainless Steel Container (Robot coupé, Inc, Ridgeland, USA) was used. Using a Milli-Q filtration system (Milli-Q from Millipore, USA), ultra-pure water was created. AFs standard product (product number CRM46304 Lot no. XA26847V with concentration of total AFs 2.6 ng/µL), OTA standard product (product number CRM46912 Lot no. LRAD1407 with concentration approximately 50 ng/µL in benzene: acetic acid (99:1), ampoule of 1 mL) and ZEN standard product ( product number CRM46916 Lot no.XA20006V with concentration 50 ng/µL in Acetonitrile, ampoule of 1 mL) were purchased from Supelco (Merck, Darmstadt, Germany).

### Aflatoxins (afs)

Methanol/water solutions (80%, 70%, 60%, 20%) were prepared to extract AFs out of the samples. AflaTest Developer solution was prepared by mixing 45 mL of filtered water and 5 mL of AflaTest Developer concentrate. 10% Tween-20, 10X concentrate PBS, 10X concentrate 0.01% Tween-20 and 10X concentrate 0.1% Tween-20 solutions were prepared by adding 10 mL from each to 90 mL distilled water. 5 X concentrate 2% Tween-20 solution was prepared by adding 20 mL to 80 mL of distilled water. ZnCl2/AL(C2H3O2) 3 was prepared by adding 25 *g* of zinc acetate to 6.25 g AlCl3 dissolved in 125 mL deionized water.

The assay was done according to the VICAM international standard guidelines (VICAM manual; https://www.vicam.com/category/aflatoxin-testing-solutions (accessed on 12 August 2023).). Briefly, a 25 *g* ground sample was weighed with 5 *g* salt (NaCl) for paprika, chili, spices, oily seeds, nuts, Gramineae, cereals, chocolate, and cocoa and placed in a blender jar. For green coffee, a 50 *g* ground sample was weighed with 5 *g* salt (NaCl). For dried fruits, dried figs, and dates, 25 *g* ground samples were weighed with no NaCl added. Then 100 mL methanol: water (80%) for chili, paprika, spices, Gramineae, cereals, and green coffee, 125 mL methanol: water (60%) for oily seed and nuts, 100 mL methanol: water (70%) for dried fruits, dried figs and dates and 100 mL of absolute methanol for chocolate and cocoa were added to the jar. The blender jar was then covered and blended for one minute at a high speed. 5 mL of filtered extract was then diluted with 20 mL purified water in case of paprika, chili, Gramineae, cereals, or green coffee or diluted with 20 mL 10% tween 20 solution in case of spices and mixed well.

For oily seeds and nuts, 20 mL of filtered extract was diluted with 20 mL of purified water. For dried fruits, dried figs, and dates, 5 mL of filtered extract was diluted with 20 mL 0.01% Tween/PBS solution and mixed well. For chocolate and cocoa, 5 mL of filtered extract was mixed with 20 mL of ZnCl2/Al(C2H3O2)3 solution. Then, using marks on the barrel to quantify 4 mL, the diluted extract was filtered through a 1.5 m microfiber filter into a clean vessel or straight into a glass syringe barrel. At a rate of approximately 1 drop/second, 4 mL of filtered diluted extract was completely passed through an AflaTest column (4 mL = 0.2 *g* sample equivalent) for chili and paprika, but for the other types of samples, 10 mL were passed through the column. Then,10 mL of methanol: water (20%) was passed through the column at a rate of about 1–2 drops/second in the case of paprika, chili, chocolate, and cocoa or 10 mL of distilled water was passed in case of spices, oily seeds, nuts, Gramineae and cereals, green coffee. The previous step was repeated once more until air went through the column. For dried fruits, dried figs, and dates, 10 mL of 0.01% Tween/PBS solution was passed through the column at a rate of 1–2 drops/second then 10 mL of purified water was passed through the column. After that, a glass cuvette was placed under the column and 1 mL HPLC grade methanol was added into a glass syringe barrel. By allowing methanol to run through the AflaTest column and collecting all the samples eluate in a glass cuvette, the column was eluted at a rate of 1 drop/second or slower. 1 mL of AflaTest Developer solution was added to eluate in the cuvette and thoroughly mixed after that. Then the cuvette was immediately placed in a calibrated fluorometer Series 4EX Fluorometer 110 V, U.S.A. (Part Number/N G8001) and 220 V, International (P/N G8002). Total AF concentration was read after 60 s.

### Ochratoxin A (OTA)

Methanol/water solutions (80%, 60%) were prepared to extract OTA out of the samples. Methanol: 1% Sodium bicarbonate solution was also prepared as previously reported. This test was performed using the VICAM international standard guidelines (https://www.vicam.com/category/ochratoxin-testing-solutions (accessed on 12 August 2023). The remaining solutions were prepared as mentioned previously for AFs. The assay was done according to the manufacturer’s manual (VICAM manual for OTA) as follows: 50 *g* ground samples were weighed with 5 *g* salt (NaCl) for paprika, chili, and spices and placed in the blender jar. But for Gramineae and cereals, 50 *g* ground samples were weighed with no NaCl added. For green coffee, roasted coffee, and Nescafe, 25 *g* ground samples were weighed with no NaCl added. Aliquots of 100 mL methanol: water (80%) for paprika, chili, spices, Gramineae, and cereals, 50 mL methanol:1% sodium bicarbonate (70%) for green and roasted coffee were added to the jar. The blender jar was covered and blended for 1 min at high speed, then 5 mL of filtered extract was diluted with 20 mL purified 10% tween 20 in case of paprika and spices and mix well. For Gramineae and cereals, 5 mL of filtered extract was diluted with 20 mL of purified 10% PBS solution. For green roasted coffee and Nescafe, 5 mL of the filtered extract was diluted with 20 mL 2% Tween-20/PBS. After that, the diluted extract was filtered through a 1.5 m microfiber filter into a clean vessel or straight into a glass syringe barrel using markings to measure 10 mL on the barrel. Then, 10 mL of filtered diluted extract were entirely run through the Ochratest column at a rate of around 1 drop/second until air went through the column (10 mL = 1.0 *g* sample equivalent).

The column was then cycled through with 10 mL of 10% PBS at a rate of roughly 1–2 drops per second in the case of paprika, chili, and spices. The previous step was then repeated once more until air went through the column. For Gramineae and cereals, 10 mL of 10% PBS was passed through the column at a rate of 1–2 drops/second then 10 mL of purified water was passed until air went through the column. For green coffee, 10 mL of 2% Tween-20/PBS was passed through the column at a rate of about 1–2 drops/second then 10 mL of purified water was passed until air came through the column. For roasted coffee and Nescafé, 10 mL of 2% Tween‐20/PBS were passed through the column at a rate of about 1–2 drops/second then 5 mL of 20%methanol\water were passed through the column at a rate of about 1–2 drops/second then 5 mL of 20% methanol\water were passed until air came through the column. Then a glass cuvette (VICAM part # 34,000) was placed under the OchraTest column and the glass syringe barrel was filled with the OchraTest Elution Solution. The column was eluted at a rate of one drop per second and all the sample eluate (1.5 mL) was collected in the glass cuvette. The cuvette was mixed and then placed into the previously mentioned fluorometer. OTA concentration was read after 60 s.

### Zearalenone (ZEN)

This test was performed using the VICAM international standard guidelines https://www.vicam.com/category/zearalenone-testing-solutions (accessed on 12 August 2023). Methanol/water solution (80%) and acetonitrile/water solution (90%) were prepared to extract ZEN from the sample. To prepare Dilute ZearalaTest Developer Solution, aluminum chloride hexahydrate was dissolved in 50 mL of HPLC Grade-methanol prior to use. The dissolved zearalatest developer was stored at room temperature for up to one month. The remaining solutions were prepared as mentioned previously for AFs and OTA. For Gramineae, 20 *g* ground samples were weighed with 2 *g* salt (NaCl) and put in a blender jar. Then 50 mL of either acetonitrile: water (90%) or methanol: water (80:20%) were added to the jar. The blender jar was then covered and blended for 2 min at high speed. 5 mL of filtered extract were then diluted with 20 mL 0.1% tween PBS buffer and then mixed well. Following that, the diluted extract was filtered through a 1.5 m microfiber filter into a clean container or straight into a glass syringe barrel utilizing markings on the barrel to measure 10 mL. These 10 mL of filtered diluted extract were entirely run through a zearalatest column at a rate of around 1 drop/second until air passed through the column (10 mL = 0.8 *g* sample equivalent). The column was then cycled through with 10 mL of 0.1% tween PBS buffer at a rate of around 1–2 drops/second until air flowed through the column then, 10 mL of deionized, or distilled water was run through it at a rate of around 2 drops per second. Finally, 1.0 mL of HPLC-grade methanol was injected into a glass syringe barrel while a glass cuvette (VICAM part # 34,000) was positioned beneath the ZearalaTest column. All the sample eluate (1 mL) from the column was collected in a glass cuvette after it was eluted at a rate of 1 drop/second. To eluate in the cuvette, 1.0 mL of ZearalaTest Developer solution was added. After thoroughly mixing the cuvette, it was immediately placed into the previously mentioned fluorometer, and the ZEN concentration was measured after 300 s.

### Standard preparation and spiking

Using certified Iso17034 and traceable to NIST (National Institute of Standards and Technology) standards, the AFs mix standard solution was prepared at 10 µg/kg while OTA standard was prepared at 20 µg/kg. ZEN standard solution was prepared at 50 µg/kg. In addition, blank (samples known to be zero MTs) samples were spiked with those prepared standard solutions at each run to be employed as quality control to guarantee accurate assessment of the data quality for all targeted MTs in regular sample analysis.

### Statistical analysis

Data was analyzed using Excel 365, Minitab 20 and SPSS 26. Count and percentage were calculated for qualitative variables while for quantitative variables, mean, SD, SE, median, first quartile, third quartile, and inter quartile range were determined as descriptive statistics. Before performing any statistical analyses, the data was cleaned. Missing information and typographical issues have been examined. Inferential statistics has been used to find correlations and differences among different food and beverage categories or type and source (export, import or local). All parametric assumptions for quantitative variables have been examined, and when necessary, the best approach was used to apply the Box-Cox transformation for non-normal dependent variables. The various models matched the data well, and following data transformation, all analyses had linear attitudes in the normal residual probability plots. *p*-values were considered significant at α < 0.05. Qualitative variables have been tested using X^2^ test, The General Linear Model’s quantitative variables were evaluated using the Student’s t test and One Way ANOVA. Post hoc analysis using Tukey was done after ANOVA where groups sharing similar letters had no significant differences. Bar charts were graphed showing percentages of each level within each category using Excel 365.

## Results

### Tested samples

**Fig. 1:**
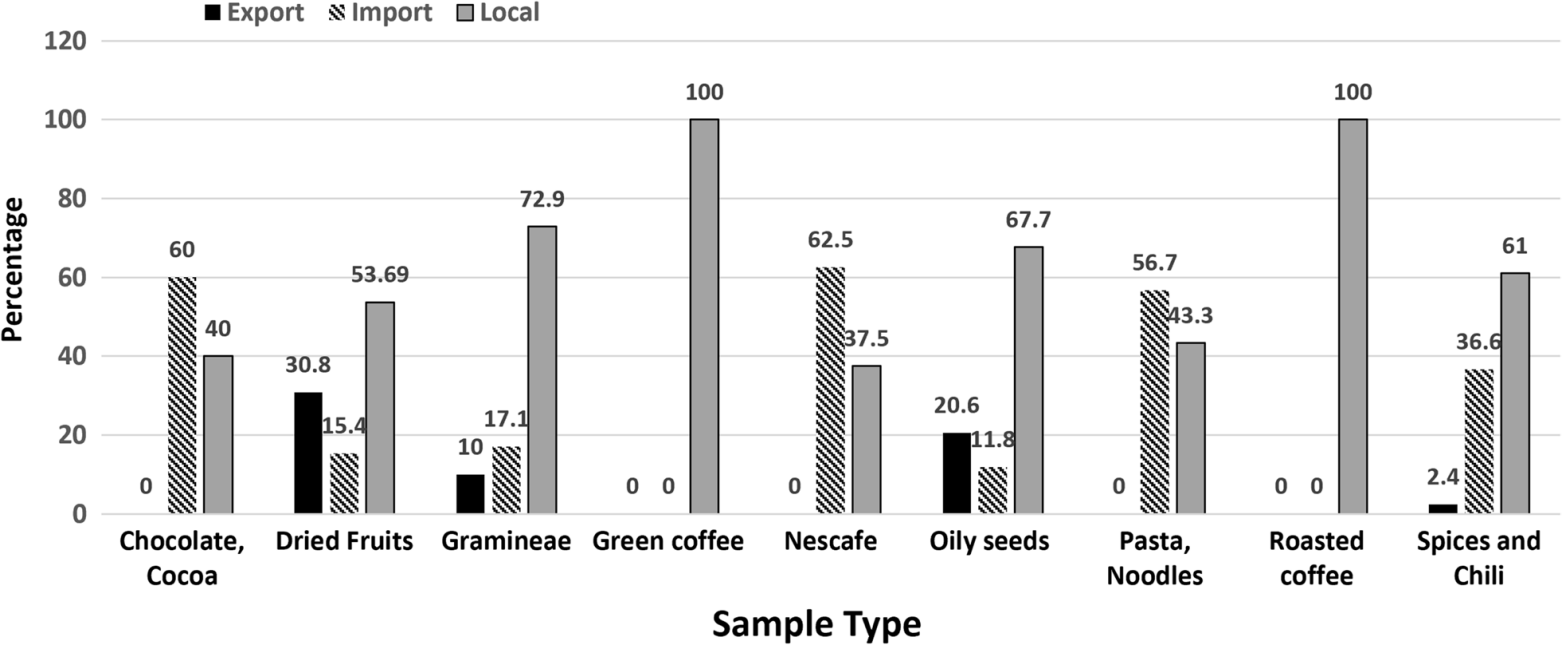
The percentage and types of various food samples collected in this study

Table 1 summarizes the number of exported, imported and local samples, the parameters tested, and the method used for each food category. Figure 1 shows the percentages of exported, imported, and local sources for each food category. All the collected samples (246 samples) were analyzed for the detection and quantification of four major MTs, including AFs; (196 samples), OTA (39), ZEN (70), and DON (100) using the ELISA followed by immunoaffinity fluorometric analysis. The food sample categories were, 19 exported, 73 imported, and 154 local samples (Table 1).

**Table 1:** A total of 246 different food samples collected from the Egyptian market during 2022

Item	Number of exported samples	Number of imported Samples	Number of locally sourced samples	Total number of examined samples for each type	Analytical Parameter
Chocolate, cocoa	0	18	12	30	AFs
Dried fruits	4	2	7	13	AFs
Gramineae (wheat, rice, oat, pediatric cereals)	7	12	51	70	AFs, OTA, ZEN, DON
Green coffee	0	0	8	8	AFs, OTA
Nescafe	0	5	3	8	OTA
Oily seeds	7	4	23	34	AFs
Pasta, noodles	0	17	13	30	DON
Roasted coffee	0	0	12	12	OTA
Spices and chili	1	15	25	41	AFs, OTA
All	19	73	154	246	

### Spectroscopic analysis of mycotoxins using ELISA

#### Aflatoxins (afs)

A total of 196 samples were screened using ELISA followed by quantitative determination of AFs using VICAM Aflatest IAC then fluorometric method. The results of the total AFs-positive samples are presented in Table 2. In the chocolate/cocoa, Gramineae, and green coffee, no total AF contamination was detected in the imported, exported, and locally sourced samples. On the other hand, 42.9% of the exported samples of oily seeds,100% of the exported samples of dried fruits, 13.3% of the imported samples, and 8% of the local samples of chili and spices exceeded the maximum limits (4 µg/kg, 4 µg/kg, 10 µg/kg, respectively) set by EOSQC 2010 (https://www.eos.org.eg/en/standard/12561, (accessed on 8 august 2023) for total AFs. Tables 3 and 4, showed significant differences among different categories of chocolate, dried fruits, and oily seeds with p-value < 0.05 while no significant difference was observed among different categories of Gramineae and spices (p-value > 0.05).

**Table 2:** Number and percentages of positive samples exceeding upper limits set by EOSQC for AFs

Type		Category	Number (percentage) of positive samples	Number (percentage) of negative samples	*P-value*
Chocolate, cocoa		Export	NA	NA	
		Import	0 (0%)	18 (100%)	
		Local	0 (0%)	12 (100%)	
Dried fruits		Export	4 (100%)	0 (0%)	0.002*
		Import	0 (0%)	2 (100%)	
		Local	0 (0%)	7 (100%)	
Gramineae		Export	0 (0%)	7 (100%)	
		Import	0 (0%)	12 (100%)	
		Local	0 (0%)	51 (100%)	
Green coffee		Export	NA	NA	
		Import	NA	NA	
		Local	0 (0%)	8 (100%)	
1Oily seeds		Export	3 (42.9%)	4 (57.1%)	0.002*
		Import	0 (0%)	4 (100%)	
		Local	0 (0%)	23 (100%)	
Spices and chili		Export	0 (0%)	1 (100%)	0.813
		Import	2 (13.3%)	13 (86.7%)	
		Local	2 (8%)	23 (92%)	

**Table 3:** One Way ANOVA results of AFs concentrations using ELISA among different categories of each type

AFs (ELISA)	Category	Mean	SE	SD	Mini	Q1	Median	Q3	Max	IQR
Chocolate, cocoa	Export	NA	NA	NA	NA	NA	NA	NA	NA	NA
	Import	0.11^B^	0.03	0.11	0.00	0.00	0.10	0.20	0.30	0.20
	Local	0.47^ A^	0.12	0.42	0.00	0.13	0.30	0.98	1.10	0.85
Dried fruits	Export	7.58^ A^	0.46	0.92	6.20	6.65	8.00	8.08	8.10	1.43
	Import	0.15^B^	0.05	0.07	0.10	*	0.15	*	0.20	*
	Local	0.83^B^	0.17	0.45	0.30	0.50	0.70	1.40	1.40	0.90
Gramineae	Export	0.51^ A^	0.19	0.51	0.00	0.20	0.30	0.70	1.50	0.50
	Import	0.48^ A^	0.12	0.41	0.00	0.13	0.45	0.80	1.20	0.68
	Local	0.68^ A^	0.07	0.53	0.00	0.30	0.50	1.00	2.60	0.70
Green coffee	Export	NA	NA	NA	NA	NA	NA	NA	NA	NA
	Import	NA	NA	NA	NA	NA	NA	NA	NA	NA
	Local	0.41	0.15	0.43	0.00	0.03	0.30	0.88	1.10	0.85
Oily seeds	Export	6.76^ A^	3.43	9.09	0.00	0.00	0.70	19.00	20.00	19.00
	Import	0.08^B^	0.08	0.15	0.00	0.00	0.00	0.23	0.30	0.23
	Local	0.64^B^	0.13	0.61	0.00	0.10	0.40	1.20	2.00	1.10
Spices and chili	Export	5.00^ A^	*	*	5.00	*	5.00	*	5.00	*
	Import	5.10^ A^	2.33	9.01	0.20	1.30	1.70	3.20	30.00	1.90
	Local	3.57^ A^	1.56	7.78	0.20	0.65	1.40	2.20	33.00	1.55

Groups that share similar letters (A or B) represent non-significant differences between different categories (local, imported and exported) while different letters (A and B) represent significant differences between categories. NA represents non-applicable category

**Table 4:** One Way ANOVA results of AFs concentrations using Fluorometer among different categories of each type

AFs (fluorometer)	Category	Mean	SE	SD	Mini	Q1	Median	Q3	Max	IQR
Chocolate, cocoa	Export	NA	NA	NA	NA	NA	NA	NA	NA	NA
	Import	0.16^B^	0.05	0.19	0.00	0.00	0.10	0.30	0.60	0.30
	Local	0.42^ A^	0.14	0.48	0.00	0.00	0.20	0.98	1.20	0.98
Dried fruits	Export	7.80^ A^	0.80	1.59	5.50	6.13	8.35	8.93	9.00	2.80
	Import	0.20^B^	0.20	0.28	0.00	*	0.20	*	0.40	*
	Local	0.61 ^B^	0.28	0.73	0.00	0.00	0.50	0.90	2.00	0.90
Gramineae	Export	0.80^ A^	0.24	0.64	0.20	0.40	0.50	1.00	2.10	0.60
	Import	0.68^ A^	0.14	0.49	0.00	0.10	0.75	1.15	1.30	1.05
	Local	0.80^ A^	0.11	0.76	0.00	0.20	0.60	1.10	3.10	0.90
Green coffee	Export	NA	NA	NA	NA	NA	NA	NA	NA	NA
	Import	NA	NA	NA	NA	NA	NA	NA	NA	NA
	Local	0.56	0.16	0.46	0.10	0.20	0.35	1.08	1.20	0.88
Oily seeds	Export	6.56^ A^	3.48	9.20	0.00	0.20	0.50	17.00	22.00	16.80
	Import	0.13^B^	0.08	0.15	0.00	0.00	0.10	0.28	0.30	0.28
	Local	0.41^B^	0.11	0.50	0.00	0.01	0.20	0.70	2.00	0.69
Spices and chili	Export	3.00^ A^	*	*	3.00	*	3.00	*	3.00	*
	Import	5.15^ A^	2.20	8.54	0.00	1.40	2.00	3.00	27.00	1.60
	Local	4.04^ A^	1.73	8.66	0.00	0.75	2.00	2.75	35.00	2.00

Groups that share similar letters (A or B) represent non-significant differences between different categories(local, imported and exported) while different letters(A and B) represent significant differences between categories. NA represents non-applicable category

#### Ochratoxin A (OTA)

Concerning OTA, a total of 139 samples were analyzed using ELISA followed by quantitative determination of OTA using VICAM Ochratest IAC and then the fluorometric method. The number and percentage of positive samples for different food types and categories are summarized in Table 5. The data indicated that green coffee, roasted coffee, and Nescafé were free of OTA contamination in the imported, exported, and locally sourced categories, while in the locally sourced samples of Gramineae, a small percentage (3.9%) showed contamination with OTA that exceeded the maximum limit set by EOSQC (3 µg/kg), but no contamination was observed in the imported and exported category. A total of 4% of spices and chili samples that are locally sourced exceeded the maximum limit set by EOSQC (15 µg/kg) but the imported and exported categories of chili and spices were free of OTA. Tables 6 and 7, showed significant differences among different categories of chocolate, dried fruits, and oily seeds with p-value < 0.05 while no significant difference was shown among different categories of Gramineae and spices(p-value > 0.05).

**Table 5:** Number and percentages of positive samples exceeding upper limits set by EOSQC for OTA

Type	Category	N (percentage) of positive samples	N (percentage) of negative samples	*p-value*
Gramineae	Export	0 (0%)	7 (100%)	0.681
	Import	0 (0%)	12 (100%)	
	Local	2 (3.9%)	49 (96.1%)	
Green coffee	Export	NA	NA	
	Import	NA	NA	
	Local	0 (0%)	8 (100%)	
Nescafe	Export	NA	NA	
	Import	0 (0%)	5 (100%)	
	Local	0 (0%)	3 (100%)	
Roasted coffee	Export	NA	NA	
	Import	NA	NA	
	Local	0 (0%)	12 (100%)	
Spices and chili	Export	0 (0%)	1 (100%)	0.000*
	Import	0 (0%)	15 (100%)	
	Local	1 (4%)	24 (96%)	

**Table 6:** One Way ANOVA results of OTA concentrations using ELISA among different categories of each type

OTA (ELISA)	Category	Mean	SE	SD	Mini	Q1	Median	Q3	Max	IQR
Gramineae	Export	0.74A	0.24	0.62	0.10	0.20	0.70	0.90	2.00	0.70
	Import	0.14A	0.04	0.15	0.00	0.00	0.10	0.30	0.40	0.30
	Local	0.67A	0.15	1.09	0.00	0.00	0.40	0.80	6.00	0.80
Green coffee	Export	NA	NA	NA	NA	NA	NA	NA	NA	NA
	Import	NA	NA	NA	NA	NA	NA	NA	NA	NA
	Local	0.21	0.05	0.16	0.00	0.10	0.20	0.30	0.50	0.20
Nescafe	Export	NA	NA	NA	NA	NA	NA	NA	NA	NA
	Import	0.92A	0.21	0.47	0.40	0.45	1.00	1.35	1.50	0.90
	Local	0.33A	0.33	0.58	0.00	0.00	0.00	1.00	1.00	1.00
Roasted coffee	Export	NA	NA	NA	NA	NA	NA	NA	NA	NA
	Import	NA	NA	NA	NA	NA	NA	NA	NA	NA
	Local	0.30	0.08	0.27	0.00	0.10	0.20	0.50	0.80	0.40
Spices and chili	Export	68.00A	*	*	68.00	*	68.00	*	68.00	*
	Import	1.77B	0.27	1.05	0.60	1.00	1.80	2.40	4.50	1.40
	Local	3.85B	1.10	5.52	0.20	0.45	1.20	5.75	20.00	5.30

Groups that share similar letters (A or B) represent non-significant differences between different categories (local, imported and exported) while different letters (A and B) represent significant differences between categories. NA represents non-applicable category

**Table 7:** One Way ANOVA results of OTA concentrations using Fluorometer among different categories of each type

OTA (fluorometer)	Category	Mean	SE	SD	Mini	Q1	Median	Q3	Max	IQR
Gramineae	Export	0.26^A^	0.09	0.25	0.00	0.00	0.30	0.50	0.50	0.50
	Import	0.09^A^	0.03	0.11	0.00	0.00	0.05	0.20	0.30	0.20
	Local	0.624^A^	0.144	1.026	0	0	0.2	1	5.5	1
Green coffee	Export	NA	NA	NA	NA	NA	NA	NA	NA	NA
	Import	NA	NA	NA	NA	NA	NA	NA	NA	NA
	Local	0.26	0.07	0.18	0.10	0.10	0.20	0.40	0.60	0.30
Nescafe	Export	NA	NA	NA	NA	NA	NA	NA	NA	NA
	Import	0.22^A^	0.09	0.20	0.00	0.00	0.30	0.40	0.40	0.40
	Local	0.07^A^	0.07	0.12	0.00	0.00	0.00	0.20	0.20	0.20
Roasted coffee	Export	NA	NA	NA	NA	NA	NA	NA	NA	NA
	Import	NA	NA	NA	NA	NA	NA	NA	NA	NA
	Local	0.22	0.07	0.25	0.00	0.00	0.15	0.40	0.70	0.40
Spices and chili	Export	64.00^A^	*	*	64.00	*	64.00	*	64.00	*
	Import	2.17^B^	0.31	1.21	0.30	1.40	2.00	3.00	5.00	1.60
	Local	4.02^B^	0.93	4.63	0.00	1.15	2.30	4.55	18.00	3.40

Groups that share similar letters (A or B) represent non-significant differences between different categories(local, imported and exported) while different letters(A and B) represent significant differences between categories. NA represents non-applicable category

### Zearalenone (ZEN)

The results of ZEN contamination analysis of 70 Gramineae samples using ELISA followed by quantitative determination of ZEN using VICAM Zeralatest IAC then fluorometric method are summarized in Table 8. In the Gramineae, no ZEN contamination was detected in the exported, imported, and locally sourced categories. Additional file 1: Tables S1 and S2 (supplementary data) showed significant differences among different categories of Gramineae with p-value < 0.05.

**Table 8:** Number and percentages of positive samples exceeding upper limits set by EOSQC for ZEN

Type	Category	N (percentage) of positive samples	N (percentage) of negative samples
Gramineae	Export	0 (0%)	7 (100%)
	Import	0 (0%)	12 (100%)
	Local	0 (0%)	51 (100%)

#### Deoxynivalenol (DON)

The results of DON contamination analysis in different food categories by applying the ELISA technique only, including Gramineae and pasta/noodles, are presented in Table 9. In the Gramineae category, no DON contamination was detected in all samples categories, while for pasta and noodles, the imported samples exhibited the highest contamination rate (above the upper limit of 750 µg/kg) set by EOSQC with 17.6% of the samples testing positive for DON. (Additional file 1: Table S3 showed that there is no significant difference among different categories of Gramineae, pasta, and noodles with p-value > 0.05.

**Table 9:** Number and percentages of positive samples exceeding upper limits set by EOSQC for DON

Type	Category	N (percentage) of positive samples	N (percentage) of negative samples	*p-value*
Gramineae	Export	0 (0%)	7 (100%)	
	Import	0 (0%)	12 (100%)	
	Local	0 (0%)	51 (100%)	
Pasta, noodles	Export	NA	NA	0.11
	Import	3 (17.6%)	14 (82.4%)	
	Local	0 (0%)	13 (100%)	

## Discussion

MTs pose a significant risk to the public’s health when they contaminate food, according to JECFA (the Joint FAO/WHO Expert Committee on Food Additives). The World Health Organization (WHO) designated MTs as priority food pollutants in the System/Food Monitoring Evaluation Program (GEMS/Food). Foods and feeds for humans and animals are invariably exposed to fungal invasion from crop planting through harvest, transportation, storage, and even into the grocery store, restaurant, and home, where the product will be ready for the consumer’s final use (Drusch and Ragab 2003). However, the development of MTs is not always linked to the expansion of fungus. The mycotoxigenic capacity of a fungus within a species mostly depends on the strain of the fungus. The physical and chemical makeup of the matrix, environmental conditions (moisture, temperature), and the species and strain of the fungus all play significant roles in the production of MTs (Drusch and Ragab 2003).

The Objective of our study was to detect and quality control various food and feed supplements for the presence of various MTs, particularly those of relevant medical importance for pediatrics from different sources in the market followed by quantitative determination of the respective MTs using standard international guidelines to estimate their possible threat to public health according to estimated daily intake and hazard index.

Our results showed that nuts (oily seeds), spices, chili, and dried fruits were highly contaminated with total AFs highlighting the need for comprehensive monitoring and control strategies. Previous studies revealed how storage conditions affected AF levels in dried fruits, nuts (oily seeds) spices (Naeem et al. 2022; Obonyo and Salano 2018; Duman 2010). The efficiency of storage circumstances in regulating the levels of AFs was in the following order: cold storage > hermetic storage in a glass jar > open-air storage (Naeem et al. 2022; Duman 2010). The hermetically sealed, waterproofed structure creates an interior-modified environment that is rich in carbon dioxide and deficient in oxygen because of the respiration of the biotic components of the stored food product. Purely aerobic aflatoxigenic molds cannot grow or produce aflatoxin under such circumstances (Naeem et al. 2022). Local shops frequently store nuts and dried fruits in bulk open-air storage, which could lead to higher levels of AF contamination before they are sold. To avoid or limit unwarranted rises in aflatoxin levels during storage, it is essential to maintain the proper storage conditions. The primary environmental factors to regulate are moisture, temperature, and relative humidity to reduce the buildup of AFs during storage (Neme and Mohammed 2017). The increased fungal development in peanuts seen during a study in India was partially caused by the predominance of fabric packaging. Fabrics are not airtight, exposing nuts or other foods to moisture that can encourage the growth of mycotoxigenic fungi (Osaili et al. 2023). In addition, when comparing samples from different parts of the world, samples from Asia had the highest prevalence of AFs. The highest percentage of infected samples were found in southern Asia, perhaps because of the region’s semi-arid, warm, and dry climates, which are ideal habitats for the formation of aflatoxigenic molds (Diella et al. 2018).

Regarding OTA analysis, our results highlighted contamination levels exceeding the upper limits set by EOSQC in spices and Gramineae. Typically, tropical settings with high temperatures, humidity, and rainfall conditions used to grow spices are ideal for the growth of microorganisms (Zhao et al. 2021). These conditions are also crucial factors in producing OTA during the time of harvest, drying, and storage of Gramineae and cereals (Li et al. 2021). Previous studies have proved that the rainy season was shown to have higher contamination levels than the dry season for spices (Li et al. 2021). In another study, it was mentioned that it is usual practice to utilize vertical silos for cereal storage, sometimes with temperature control and aeration, to maintain the grain’s quality and safety (Troestch et al. 2022).

Regarding DON analysis, our results showed high contamination levels in pasta and noodles. In previous studies, DON was found in 180 samples of Chinese wheat, with varying levels from 14.52 to 41157.13 *g*/kg (mean level 488.02 *g*/kg). The humid and hot weather circumstances in Pakistan throughout the summer may be the cause of the elevated incidence levels of DON in wheat and wheat product samples (Iqbal et al. 2020). The fluctuation in toxin structure during cropping seasons determines the amounts of toxins across the seasons (Obonyo and Salano 2018). In the summer, the wheat crop is frequently harvested in May and June, which could increase the risk of a fungal infection prior to, after, or during the storage of MTs. High frequency of DON in wheat and products made from wheat may also be brought on using vulnerable wheat cultivars, outmoded traditional agricultural techniques, a lack of crop rotation, and no-till farming. In addition, people sometimes keep grains like wheat or maize in dirt bins in rural regions, which can draw moisture from the environment and cause fungus epidemics (Iqbal et al. 2014).

For the use of the ELISA method of analysis, previous studies proved that demand has increased for the creation of a method that is sensitive, accurate, quick, simple, and reliable for detection at low concentrations, such as those observed in milk samples. ELISA technique was judged to be sufficiently meeting the requirements for official control purposes for this purpose (Kos et al. 2016). Previous research in Jordan focused on reducing the amount of MTs in the domestic food supply, including the issue of reliable sampling and analysis methods. It also aimed to enhance and demonstrate the analytical capabilities of laboratories in Jordan and developing nations, enabling them to effectively monitor MTs in food and get around non-tariff barriers. To demonstrate that, the ELISA approach is appropriate for detecting MTs at extremely low concentrations, validation studies were conducted (Omar et al. 2020). Additionally, a prior article claimed that this validated method could possibly be used as a sensitive and high-throughput screening for the mycotoxin sterigmatocystin in food (Oplatowska et al. 2018). Regarding the IAC method followed by fluorometer detection, an earlier study in the Sudanese state of Khartoum used a fluorometer and the Vicam method to measure the amount of aflatoxin M1 (AFM1) contamination in raw and imported powdered milk (Ali et al. 2014). In Khartoum state, Sudan, another survey was conducted to look for the presence of aflatoxin B(1) in 60 duplicate samples (120 samples) of peanuts. The toxin was removed from the samples using an AflaTest-P affinity column, and the concentration was determined using a calibrated Vicam fluorometer. In every single one of the examined samples, different amounts of aflatoxin B (1) were found (Elshafie et al. 2011). It was reported in earlier research that combining a double-extract cleanup and a fluorometric measurement to determine the presence of OTA in red wine has the advantage of reducing both the cost and time of the study (Longobardi et al. 2013). In our study, all the collected food samples were analyzed for the four major MTs, including AF, OTA; ZEN, and DON using both ELISA and immunofluorometric methods and the obtained resulst revealed no differences could be observed in the sensitivity of both methods. However, significant differences were observed among different food categories, particularly the local and imported ones which highlighted the urgent for need strict and appropriate control measures to minimize the risk of MTs adverse effects. In conclusion, our findings confirmed non-significant differences between the two methods in the detection of AFs, OTA, ZEN, and DONin various food categories and therefore, can substitute each other whenever possible. This study highlights the need for continuous monitoring of mycotoxin contamination in various food categories, strict quality control measures during exportation and importation processes, and continued improvement of production and storage practices in locally sourced category samples to minimize the presence of MTs and ensure the quality and safety of food consumed by the general population. Further research is necessary to explore the specific sources of contamination and develop effective approaches for prevention.

### Supplementary information

**Additional file 1:** **Table S1.** One Way ANOVA results of ZEN concentrations using ELISA among different categories of each type. **Table S2.** One Way ANOVA results of ZEN concentrations using Fluorometer among different categories of each type. **Table S3.** One Way ANOVA results of DON concentrations using ELISA among different categories of each type.

## Data Availability

All data generated or analyzed during this study are included in this published article and supplementary file.
