# Supplementary material for: Evaluation of ELISA and immunoaffinity fluorometric analytical tools of four mycotoxins in various food categories
Source: AMB Express. 2023 Nov 3;13:123. doi: 10.1186/s13568-023-01629-5 (PMC10624774; doi:10.1186/s13568-023-01629-5)
Supplement: Supplementary file 1 — Additional file 1: Table S1. One Way ANOVA results of ZEN concentrations using ELISA among different categories of each type. Table S2. One Way ANOVA results of ZEN concentrations using Fluorometer among different categories of each type. Table S3. One Way ANOVA results of DON concentrations using ELISA among different categories of each type. [file 13568_2023_1629_MOESM1_ESM.docx]

**Table S1**. One Way ANOVA results of ZEN concentrations using ELISA among different categories of each type.

| ZEN (ELISA) | Source | Mean |  | SE | SD | Mini | Q1 | Median | Q3 | Max | IQR |
| --- | --- | --- | --- | --- | --- | --- | --- | --- | --- | --- | --- |
| Gramineae | Export | 13.21^A^ |  | 1.45 | 3.83 | 6.00 | 11.00 | 14.50 | 15.00 | 18.00 | 4.00 |
|  | Import | 6.98^B^ |  | 1.28 | 4.43 | 1.60 | 2.75 | 6.10 | 10.00 | 16.00 | 7.25 |
|  | Local | 6.79^B^ |  | 0.55 | 3.95 | 0.50 | 3.60 | 6.00 | 11.00 | 14.00 | 7.40 |

Groups that share similar letters are non-significant while different letters represent significant differences.

**Table S2**. One Way ANOVA results of ZEN concentrations using Fluorometer among different categories of each type.

| ZEN (fluorometer) | Source | Mean | SE | SD | Mini | Q1 | Median | Q3 | Max | IQR |
| --- | --- | --- | --- | --- | --- | --- | --- | --- | --- | --- |
| Gramineae | Export | 11.59A | 0.91 | 2.40 | 7.00 | 10.00 | 12.00 | 13.60 | 14.00 | 3.60 |
|  | Import | 6.94AB | 1.43 | 4.94 | 1.60 | 2.25 | 5.70 | 11.78 | 15.20 | 9.53 |
|  | Local | 6.53B | 0.59 | 4.23 | 0.00 | 3.00 | 5.80 | 9.00 | 15.00 | 6.00 |

Groups that share similar letters are non-significant while different letters represent significant differences.

**Table S3**. One Way ANOVA results of DON concentrations using ELISA among different categories of each type.

| **DON (ELISA)** | **Source** | **Mean** | **SE** | **SD** | **Mini** | **Q1** | **Median** | **Q3** | **Max** | **IQR** |
| --- | --- | --- | --- | --- | --- | --- | --- | --- | --- | --- |
| Gramineae | Export | 34.57^A^ | 6.83 | 18.06 | 19.00 | 20.00 | 30.00 | 57.00 | 63.00 | 37.00 |
|  | Import | 29.09^A^ | 7.43 | 25.73 | 4.50 | 9.07 | 16.50 | 53.25 | 77.00 | 44.17 |
|  | Local | 27.74^A^ | 3.96 | 28.31 | 1.2 | 8.9 | 15 | 40 | 100 | 31.1 |
| Pasta, Noodles | Export | NA | NA | NA | NA | NA | NA | NA | NA | NA |
|  | Import | 159.60^A^ | 72.60 | 299.20 | 4.00 | 11.30 | 30.00 | 47.80 | 795.00 | 36.50 |
|  | Local | 44.71^A^ | 5.87 | 21.16 | 12.90 | 28.50 | 44.00 | 62.50 | 81.00 | 34.00 |

Groups that share similar letters are non-significant while different letters represent significant differences. (NA) stands for this item is non-applicable.
